# Hepatitis B virus reactivation risk in haploidentical versus matched donor transplantation: Impact of universal prophylaxis

**DOI:** 10.1016/j.htct.2026.106519

**Published:** 2026-09-05

**Authors:** Tzu-Ting Chen, Su-Peng Yeh, Ching-Chan Lin

**Affiliations:** a Division of Hematology and Oncology, Department of Internal Medicine, China Medical University Hospital, China Medical University, Taiwan College of Medicine, Taichung, 404, Taiwan; b Graduate Institute of Biomedical Sciences, College of Medicine, China Medical University, Taichung, Taiwan, 40447

**Keywords:** Hepatitis B virus, Hematopoietic cell transplantation, Haploidentical transplant, Antiviral prophylaxis, Post-transplant cyclophosphamide

## Abstract

**Background:**

Hepatitis B virus reactivation is a serious complication of allogeneic hematopoietic cell transplantation, particularly in endemic regions. Patients with HBsAg-negative/anti-HBc-positive serology represent an intermediate-risk group due to occult hepatitis B virus infection. This study evaluated hepatitis B virus reactivation risk in haploidentical versus matched donor hematopoietic cell transplantation and assessed the effectiveness of Taiwan's universal prophylaxis policy.

**Methods:**

A retrospective cohort study of 165 adult hematopoietic cell transplantation recipients with HBsAg-negative/anti-HBc-positive status was conducted at China Medical University Hospital, Taiwan from 2010–2025. The incidence of hepatitis B virus reactivation was compared between the pre-prophylaxis (2010–February 2021) and post-policy implementation (March 2021–2025) periods. Univariable associations, clinical outcomes, and temporal patterns were analyzed.

**Results:**

Pre-prophylaxis, haploidentical recipients had higher reactivation rates (11.2%vs. 2.9%), but this did not reach statistical significance due to the small number of events (Odds ratio: 4.31; 95% CI: 0.67–37.55; p = 0.279). In the univariable analyses, hepatitis B virus reactivation was associated with chronic graft-versus-host disease (p = 0.001) and donor anti-HBs negativity (p = 0.001), with a median time to reactivation of 8.2 months.

**Conclusion:**

In this cohort, haploidentical hematopoietic cell transplantation with post-transplant cyclophosphamide was associated with a higher observed rate of hepatitis B virus reactivation than matched donor transplantation in HBsAg-negative/anti-HBc-positive recipients, although this difference was not statistically significant.

## Introduction

Hematopoietic cell transplantation (HCT) is a potentially curative therapy for various hematologic malignancies but remains associated with serious complications, including viral reactivations. Hepatitis B virus (HBV) reactivation has long been a major concern in allogeneic HCT, occurring in 45%–86% of HBsAg‑positive recipients who do not receive prophylaxis, and can lead to graft failure, hepatic complications, and transplant-related mortality exceeding 30% [1, 2, 3]. Over the past two decades, routine antiviral prophylaxis has substantially reduced reactivation, rendering HBV reactivation largely preventable in contemporary practice [1, 2, 3].

Recipients who are HBsAg‑negative/anti‑HBc‑positive constitute a distinct intermediate‑risk group increasingly recognized in transplant medicine [4, 5, 6]. This serologic profile, which reflects resolved or prior HBV infection with occult persistence of covalently closed circular DNA, occurs in 15%–20% of adults in HBV-endemic regions such as Taiwan; reported reactivation rates range from 5% to 40%, depending on the intensity of immunosuppression [4, 5, 6]. Accumulating data and contemporary international guidelines from the American Gastroenterological Association (AGA), American Association for the Study of Liver Diseases (AASLD), American Society of Clinical Oncology (ASCO), and European Conference on Infections in Leukaemia (ECIL) now classify allogeneic HCT as high risk for HBV reactivation and recommend antiviral prophylaxis for HBsAg‑negative/anti‑HBc‑positive recipients [7, 8, 9, 10, 11]. The 2025 AGA guidelines and ECIL recommendations further support prolonged prophylaxis beyond immunosuppression until immune reconstitution. Entecavir prophylaxis has been shown to reduce reactivation to 2.4% versus 13.7% with preemptive monitoring [10, 11, 12].

Haploidentical HCT with post‑transplant cyclophosphamide (PTCy) has expanded donor availability by overcoming HLA barriers but confers distinct infectious risks [13, 14, 15, 16]. PTCy‑based platforms are associated with higher rates of viral reactivation, including cytomegalovirus, Epstein-Barr virus, and human herpesvirus‑6, likely due to prolonged immunosuppression, delayed T‑cell recovery, and impaired antigen‑specific responses [13, 14, 15, 16]. However, data on HBV reactivation in haploidentical recipients, particularly those who are HBsAg‑negative/anti‑HBc‑positive, remain limited. Taiwan offers a distinctive HBV‑endemic setting with long‑standing universal HBV vaccination and national antiviral coverage; before March 2021, prophylaxis for HBsAg‑negative/anti‑HBc‑positive HCT recipients was inconsistent, with universal antiviral prophylaxis for this group only being adopted under the National Health Insurance program in March 2021 [12].

Against this background, this study aimed to compare HBV reactivation rates between haploidentical and matched donor HCT in HBsAg‑negative/anti‑HBc‑positive recipients, evaluate the real‑world impact of universal antiviral prophylaxis, and identify clinical predictors of reactivation. In this study, HBV reactivation in HBsAg‑negative/anti‑HBc‑positive recipients was defined in line with the updated ECIL‑9 guidance, which upholds the ECIL‑5 criteria and defines reactivation as detection of HBsAg in individuals previously HBsAg‑negative, detection of HBV DNA in patients previously negative for HBV DNA, or a ten‑fold increase in HBV DNA levels in patients with previously detectable HBV DNA; these criteria are concordant with those of other international societies [9, 10, 11].

## Methods

### Study design and population

This retrospective single-center cohort study was conducted at China Medical University Hospital, Taiwan, between January 2010 and March 2025 with approval of the institutional review board (IRB: CMUH111-REC3–034). Adults (≥18 years) with hematologic malignancies who underwent allogeneic peripheral blood stem cell transplantation (PBSCT) and were HBsAg-negative/anti-HBc-positive were included; patients with HBsAg positivity, viral co-infections, cord blood transplantation, incomplete serologic data, or who declined to participate were excluded.

### Transplant protocols

Haploidentical transplantation was defined as transplantation from a first-degree relative with ≥2 HLA mismatches at HLA-A, -B, -C, or -DRB1, utilizing post-transplant cyclophosphamide (PTCy) 50 mg/kg on Days +3 and +4, followed by a calcineurin inhibitor and mycophenolate mofetil starting on Day +5. Matched donor recipients received standard calcineurin inhibitor-based graft-versus-host disease (GVHD) prophylaxis, with conditioning classified as myeloablative or reduced-intensity according to established criteria.

### Hepatitis B virus monitoring and prophylaxis

HBsAg, anti-HBc, and anti-HBs were measured by chemiluminescent immunoassay, and HBV DNA by real-time polymerase chain reaction (PCR - lower limit of detection 20 IU/mL); anti-HBs ≥10 IU/L was considered positive. During the study period, there was no fixed protocol-defined interval for post-transplant HBV monitoring; instead, the timing and frequency of HBsAg and HBV DNA assessments were left to the discretion of the treating physician guided by routine clinical follow-up, liver biochemistry, and the overall immunosuppressive status of the patient. Before March 2021, antiviral prophylaxis was given at physician discretion, whereas thereafter all HBsAg-negative/anti-HBc-positive recipients received entecavir (0.5 mg) or tenofovir disoproxil fumarate (300 mg) once daily for at least 12 months post-transplant.

### Endpoints and definitions

The primary endpoint was the cumulative incidence of HBV reactivation. In accordance with the updated ECIL‑9 recommendations, which uphold the ECIL‑5 definition, HBV reactivation was defined by any of the following criteria: (i) detection of HBsAg in individuals who were previously HBsAg‑negative; (ii) detection of HBV DNA in patients who were previously negative for HBV DNA; or (iii) a ten‑fold increase in HBV DNA levels in patients with previously detectable HBV DNA. These criteria are consistent with those endorsed by other major international societies for patients under immunosuppression at risk of HBV reactivation. All patients included in this study were HBsAg‑negative and had undetectable HBV DNA at baseline.

### Statistical analysis

Categorical variables were compared using chi-square tests and continuous variables using the Mann-Whitney U test. Cumulative incidence was estimated by Kaplan-Meier methods with Fine-Gray competing risk models for transplant-related mortality. Given the limited number of HBV reactivation events, baseline variables associated with HBV reactivation were explored using univariable comparisons between patients with and without reactivation. Analyses were stratified into pre-policy (2010–February 2021) and post-policy (March 2021–2025) periods and performed using SPSS v28.0 and R v4.2.0, with a two-sided p < 0.05 considered statistically significant.

## Results

### Study population

In total, 165 HBsAg-negative/anti-HBc-positive adults underwent allogeneic PBSCT between 2010 and 2025. Before March 2021, 115 patients were transplanted without universal prophylaxis, whereas 50 patients received HCT after policy implementation; median follow-up was 62.3 months and 18.4 months, respectively. Patient characteristics stratified by reactivation status are summarized in Table 1. HBV reactivation occurred in ten patients (8.7%), indicating a notable clinical risk in this population.

**Table 1:** Clinical characteristics of allogeneic peripheral blood stem cell transplantation recipients, stratified by HBV reactivation status (pre-March 2021).

	HBV reactivation after allogeneic PBSCT (n = 10)	No HBV reactivation after allogeneic PBSCT (n = 105)	p-value
Age (years) - median (range)	53 (35–70)	58 (19–81)	0.475
Gender, male – n (%)	8 (80.0%)	62 (59.0%)	0.338
Type of malignancy – n (%)			
AML/MDS	6 (60.0%)	67 (63.8%)	
ALL	1 (10.0%)	23 (21.9%)	
CML/MPN	0 (0%)	3 (2.9%)	
Lymphoma	3 (30.0%)	9 (8.6%)	
MM	0 (0%)	3 (2.8%)	
Disease status before HCT – n (%)			0.870
Complete remission	7 (70.0%)	65 (61.9%)	
Active disease	3 (30.0%)	40 (38.1%)	
HCT-CI score – n (%)			
>2	4 (40.0%)	27 (25.7%)	0.549
Conditioning regimen – n (%)			0.73
MAC	2 (20.0%)	26 (24.7%)	
RIC	8 (80.0%)	79 (75.3%)	
BMT type – n (%)			0.870
MSD	0 (0%)	10 (9.5%)	
MUD	1 (10.0%)	24 (22.9%)	
Haploidentical	9 (90.0%)	71 (67.8%)	
Donor pre-HCT ant-HBs titer – n (%)			0.001
Negative	7 (70.0%)	19 (18.7%)	
Positive	3 (30.0%)	38 (36.1%)	
Unknown	0 (0%)	48 (45.7%)	
Acute GvHD – n (%)			
Any Grade	5 (50.0%)	33 (31.4%)	0.400
Chronic GvHD – n (%)			
Any Grade	7 (70.0%)	19 (18.1%)	0.001

ALL: acute lymphoblastic leukemia; AML: acute myeloid leukemia; anti-HBs: hepatitis B surface antibody; CML: chronic myeloid leukemia; GVHD: graft-versus-host disease; HCT: hematopoietic cell transplantation; HCT-CI: hematopoietic cell transplantation comorbidity index; MAC: myeloablative conditioning; MDS: myelodysplastic syndrome; MM: multiple myeloma; MPN: myeloproliferative neoplasm; MSD: matched sibling donor; MUD: matched unrelated donor; PBSCT: peripheral blood stem cell transplantation; RIC: reduced-intensity conditioning.

The median age was 53 years in the reactivation group (range: 35–70 years) versus 58 years in the non-reactivation group (range: 21–81 years), with no significant difference (p = 0.455).

Several baseline characteristics differed between patients with and without HBV reactivation. Chronic GVHD of any grade was more common in the reactivation group than in the non-reactivation group (70.0%vs. 19.0%; p = 0.001). Donor anti-HBs negativity was also more frequent among patients who developed HBV reactivation (70.0%vs. 18.1%; p = 0.001). The disease status before transplantation, Hematopoietic Cell Transplantation-Specific Comorbidity Index scores, and conditioning regimen intensity were balanced between the groups. The complete remission rates were comparable (70.0%vs. 61.9%; p = 0.870), with no significant differences in conditioning intensity or comorbidity burden.

### Donor type and risk of hepatitis B virus reactivation

**Figure 1:**
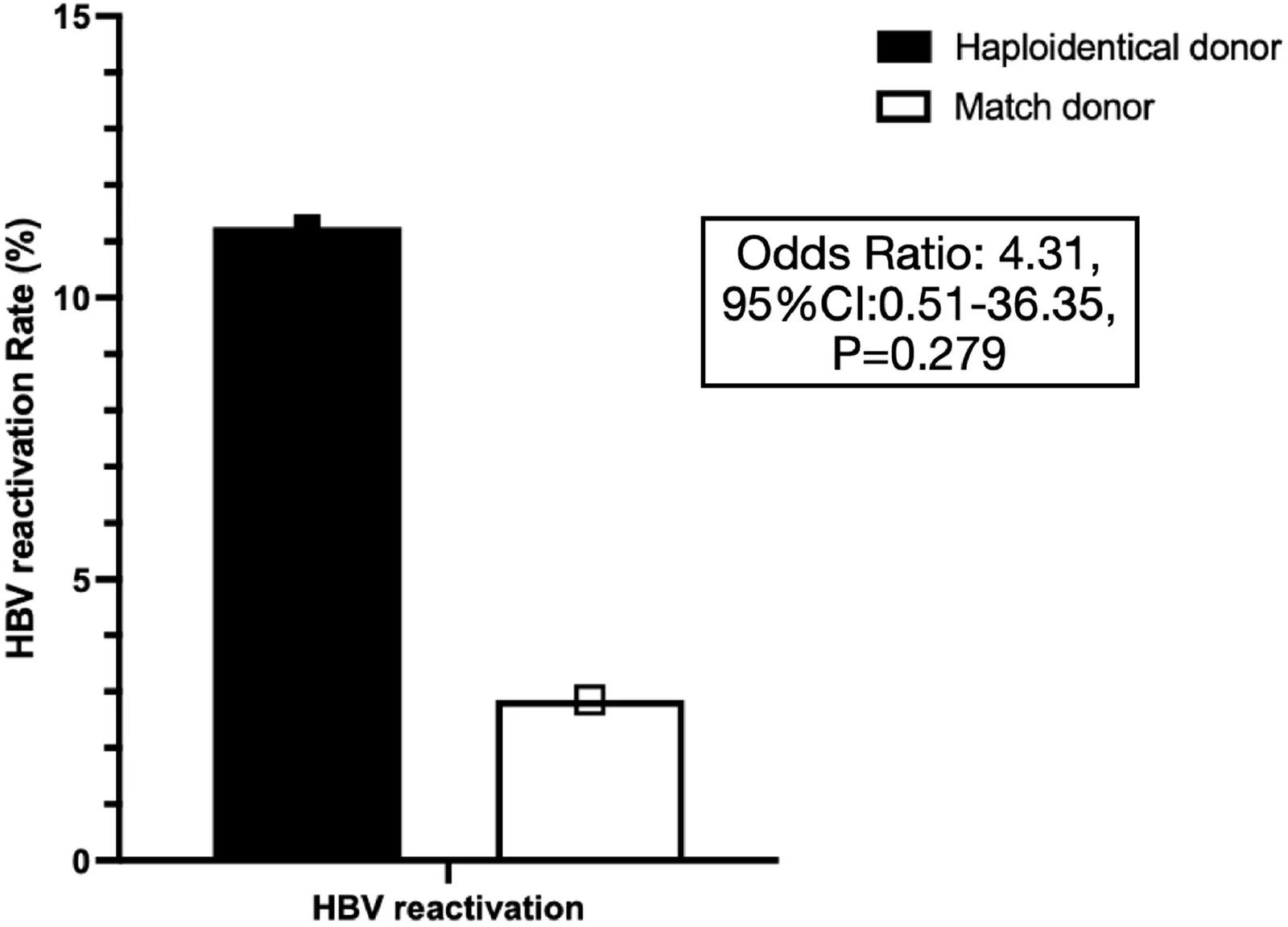
percentage of hepatitis B virus (HBV) reactivation events among HBsAg-negative/HBcAb-positive recipients undergoing haploidentical and matched allogeneic peripheral blood stem cell transplantation.

Among pre-prophylaxis recipients, HBV reactivation occurred in ten of 89 haploidentical transplants (11.2%) versus one of 34 matched donor transplants (2.9%), indicating a numerically higher observed rate in the haploidentical group; however, this difference was not statistically significant (OR 4.31; 95% CI: 0.67–37.55; p = 0.279), reflecting the small number of events in the matched donor cohort (Figure 1).

Haploidentical transplants (with post-transplant cyclophosphamide) showed a higher observed rate of HBV reactivation compared with matched unrelated or sibling donor (MUD/MSD) transplants; however, this difference did not reach statistical significance (OR: 4.31; 95% CI: 0.67–37.55; p = 0.279)

### Influence of temporal patterns and transplant platform on hepatitis B virus reactivation risk

All reactivation events fulfilled the predefined ECIL‑based criteria for HBV reactivation. The median time to HBV reactivation was 8.2 months (interquartile range: 4.1–14.6 months) post-transplant.

### Impact of antiviral prophylaxis on the reactivation rates of hepatitis B virus

**Figure 2:**
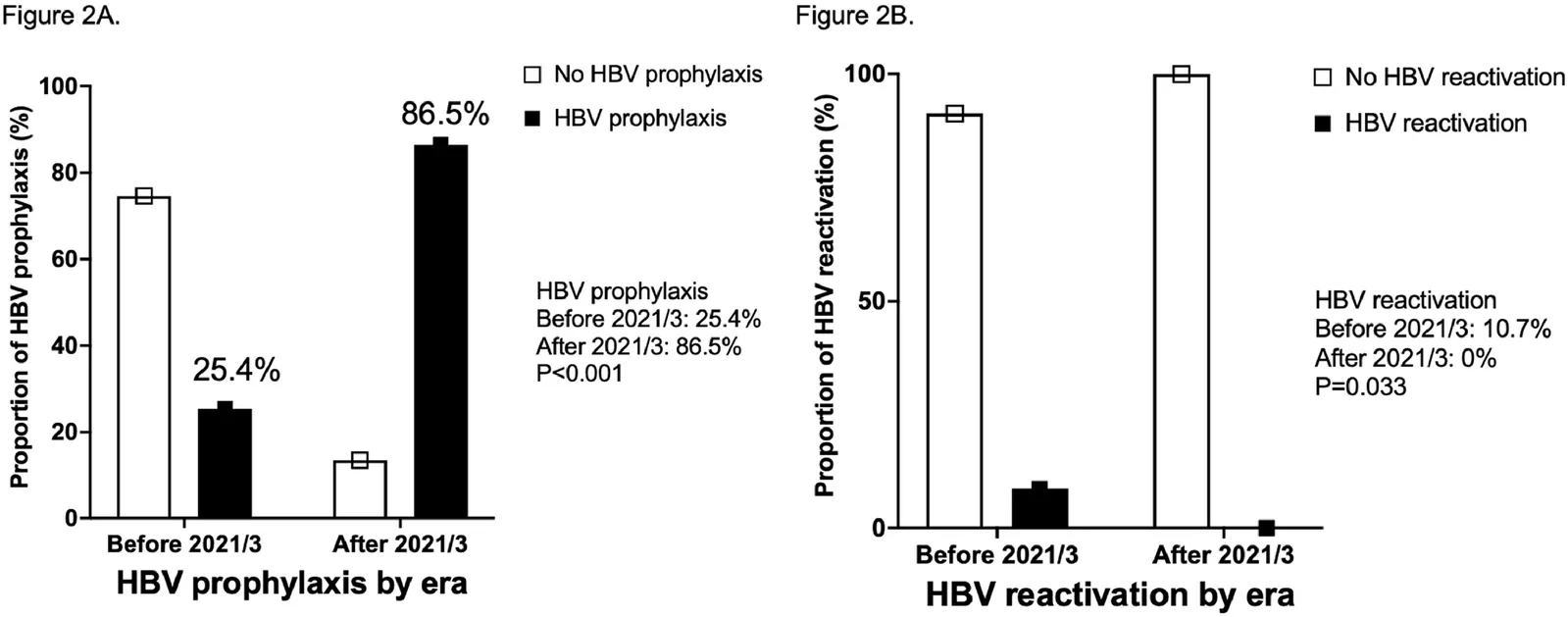
Universal prophylaxis and hepatitis B virus (HBV) reactivation. (A) Proportion of patients receiving HBV prophylaxis before and after the introduction of the reimbursement policy in March 2021. (B) HBV reactivation in the pre‑policy period (10/115; 8.7%) versus the post‑policy period (0/50; 0%).

The introduction of Taiwan’s prophylaxis policy in March 2021 resulted in major changes in clinical practice and outcomes. Under this policy, HBV prophylaxis was reimbursed from the start of SCT for approximately one year, covering the transplant period and up to six months after discontinuation of GVHD prophylaxis. Among the 165 patients spanning the entire study period (2010–2025), prophylaxis usage increased significantly from 26.9% in the pre-policy period to 80.7% post-policy (p < 0.001; Figure 2A).

Most notably, HBV reactivation rates decreased from 8.7% before universal prophylaxis (10/115 patients) to 0% after policy implementation (0/50 patients; p = 0.033; Figure 2B). Consequently, no reactivation was observed among patients who underwent transplantation after March 2021, indicating the complete elimination of reactivation in the prophylaxis period and providing compelling real-world evidence for systematic prevention.

### Survival outcomes and clinical impact

**Figure 3:**
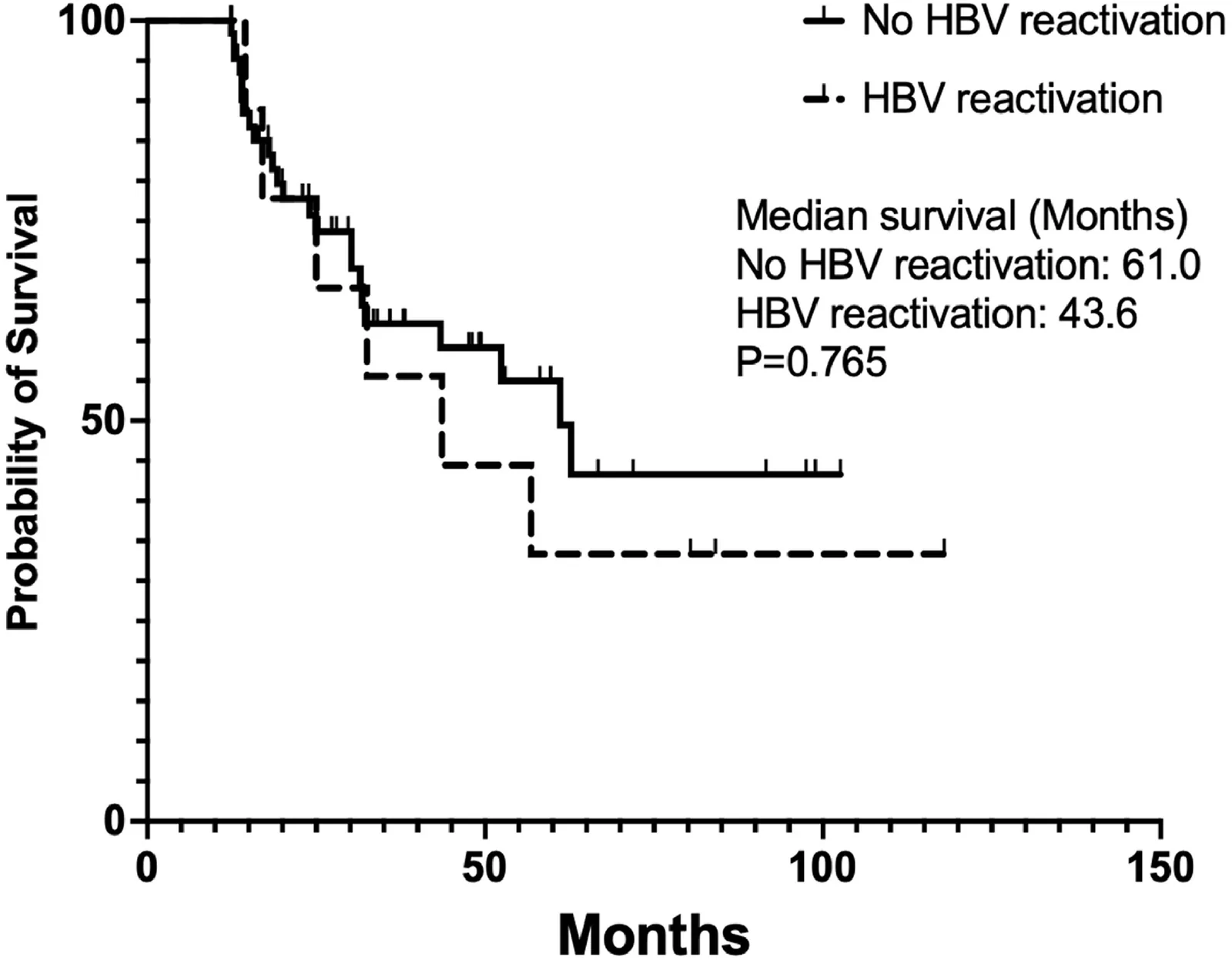
Overall survival according to hepatitis B virus (HBV) reactivation status. Kaplan–Meier curves show no significant difference in overall survival between patients with and without HBV reactivation (p = 0.765).

In the pre-prophylaxis cohort, HBV reactivation did not significantly affect long-term transplant outcomes, with Kaplan-Meier survival curves largely overlapping between groups. Patients with reactivation had a numerically lower median survival (43.6 months) than those without reactivation (61.0 months), but this difference was not significant (p = 0.765; Figure 3). These findings suggest that although HBV reactivation is clinically important and requires prompt antiviral management, timely intervention mitigated its effect on overall survival.

## Discussion

In this cohort, HBsAg‑negative/anti‑HBc‑positive patients undergoing haploidentical HCT with PTCy had a numerically higher observed rate of HBV reactivation than those receiving matched donor transplantation; however, this difference did not reach statistical significance (OR: 4.31; 95% CI: 0.67–37.55; p = 0.279) and should be interpreted as exploratory. Universal antiviral prophylaxis was associated with the elimination of observed HBV reactivation events in the post‑policy cohort [13, 14, 15, 16]. In this cohort, chronic GVHD and donor anti-HBs negativity were associated with HBV reactivation, suggesting that immune dysregulation and the absence of adoptive HBV-specific immunity may contribute to reactivation risk; however, these findings were based on univariable exploratory analyses and should not be interpreted as definitive independent risk factors. Taiwan's universal prophylaxis policy, which increased coverage from 26.9% to 80.7% and reduced observed HBV reactivation from 8.7% (10/115) to 0% (0/50), supports the value of systematic guideline-directed prophylaxis in this high-risk population [1].

Based on these findings and current international guidelines [6, 7, 8, 9, 10, 11], universal prophylaxis with entecavir or tenofovir for at least 12 months, with extended duration in patients with chronic GVHD, preferential selection or vaccination of anti‑HBs‑positive donors, and structured post‑prophylaxis HBV DNA monitoring are recommended to minimize reactivation risk.

This study has several limitations. First, it was a retrospective single‑center analysis, which may limit generalizability. Second, the number of HBV reactivation events was small, and analyses of factors associated with reactivation were limited to univariable comparisons without multivariable adjustment; therefore, these findings should be interpreted as exploratory and hypothesis‑generating rather than definitive independent risk factors. Third, although the revised definition of HBV reactivation used in this study was aligned with ECIL‑9/ECIL‑5 criteria, it differs from some AASLD 2018‑based definitions for HBsAg‑negative/anti‑HBc‑positive patients and from thresholds used in previous studies, which may affect cross‑study comparability. Fourth, post‑transplant HBV monitoring was not performed at fixed protocol‑defined intervals but was guided by physician discretion, potentially leading to under‑detection of low‑level or subclinical reactivation. Finally, follow‑up duration was substantially shorter in the post‑policy era than in the pre‑policy era, which may have reduced the detection of late HBV reactivation events after universal prophylaxis implementation.

## Funding

This study was supported in part by 10.13039/100007404China Medical University, under grant numbers ANHRF111-21 and NHRI-108A1-CACO-13191902.

## Data availability

The data that support the findings of this study are available from the corresponding author upon reasonable request.

## Author contributions

Conceptualization: Tzu-Ting Chen; Methodology: Formal analysis and investigation: Tzu-Ting Chen; Writing–original draft preparation Tzu-Ting Chen; Writing–review and editing: Ching-Chan Lin; Funding acquisition and Resources: Su-peng Yeh; Supervision: Ching-Chan Lin.

## Conflicts of interest

The authors report there are no competing interests to declare.
